# Characterization of two pathological gating-charge substitutions in Cav1.4 L-type calcium channels

**DOI:** 10.1080/19336950.2023.2192360

**Published:** 2023-03-21

**Authors:** Thomas Heigl, Michael A. Netzer, Lucia Zanetti, Matthias Ganglberger, Monica L. Fernández-Quintero, Alexandra Koschak

**Affiliations:** a University of Innsbruck, Institute of Pharmacy, Pharmacology and Toxicology, Innsbruck, Austria; b Institute of General, Inorganic and Theoretical Chemistry, Center for Chemistry and Biomedicine, University of Innsbruck, Innsbruck, Austria; c Division of Pharmacology and Toxicology, Department of Pharmaceutical Sciences, University of Vienna, Vienna, Austria

**Keywords:** L-type calcium channels, Cav1.4, calcium channelopathies, voltage sensor, congenital stationary night blindness type 2

## Abstract

Cav1.4 L-type calcium channels are predominantly expressed at the photoreceptor terminals and in bipolar cells, mediating neurotransmitter release. Mutations in its gene, *CACNA1F*, can cause congenital stationary night-blindness type 2 (CSNB2). Due to phenotypic variability in CSNB2, characterization of pathological variants is necessary to better determine pathological mechanism at the site of action. A set of known mutations affects conserved gating charges in the S4 voltage sensor, two of which have been found in male CSNB2 patients. Here, we describe two disease-causing Cav1.4 mutations with gating charge neutralization, exchanging an arginine 964 with glycine (RG) or arginine 1288 with leucine (RL). In both, charge neutralization was associated with a reduction channel expression also reflected in smaller ON gating currents. In RL channels, the strong decrease in whole-cell current densities might additionally be explained by a reduction of single-channel currents. We further identified alterations in their biophysical properties, such as a hyperpolarizing shift of the activation threshold and an increase in slope factor of activation and inactivation. Molecular dynamic simulations in RL substituted channels indicated water wires in both, resting and active, channel states, suggesting the development of omega (*ω*)currents as a new pathological mechanism in CSNB2. This sum of the respective channel property alterations might add to the differential symptoms in patients beside other factors, such as genomic and environmental deviations.

## Introduction

Cav1.4 L-type voltage-gated calcium channels are predominantly expressed in retinal photoreceptor cells [1–4], coupling light-induced membrane potential changes to neurotransmitter release at the axon terminal [5–7]. Transportation of Cav1.4 channels to their site of action and their modulation is enabled by β_2_ and α_2_δ_4_ subunits in the channel complex. Among other proteins, these auxiliary subunits also provide the synaptic environment suitable for proper signal transmission to second-order neurons [8,9]. Mutations in the gene of Cav1.4, *CACNA1F*, can lead to X-linked retinal diseases such as Åland Island Eye Disease (AIED) [10], cone-rod dystrophy (CORDX3) [11] or congenital stationary night blindness type 2 (CSNB2) [12,13]. By sequencing, the *CACNA1F* gene locus of affected patients, a wealth of mutations have been collected [14]. Substituted amino acids are mostly localized in conserved regions, crucial for channel stability and gating, as well as protein-protein interactions [15,16], however their effects have been characterized *only* for few channel mutations *in vitro* [17–22] (for review see [15]). The voltage sensor (S4 helix) belongs to these conserved regions and is found in all four voltage-sensing domains (VSDs). It consists of repeating positively charged amino acids that form salt bridges with negative counter charges from surrounding helices in the VSD [23]. Water crevices are formed on each side of the membrane, reaching toward the hydrophobic center of the VSD and focusing the electric field through the S4 helix [24–27]. Owing to their positive charges, the S4 helices can sense changes in the membrane potential and transform their relative position according to the electrical field, inducing the opening and closing of the channel pore (for review see [28]). Neutralization of these amino acids in other voltage-gated ion channels has been linked to diseases, such as hypokalemic periodic paralysis (Nav1.4 [29] or Cav1.1 [30]), normokalemic periodic paralysis (Nav1.4 [31] or Cav1.1 [32]), mixed arrhythmias associated with dilated cardiomyopathy (Nav1.5 [33]) and peripheral nerve hyperexcitability (Kv7.2 [34]). The substitution of distinct positive S4-helix residues facilitates the connection of both water-filled vestibules surrounding the VSD by providing more space in between the helices to form a water wire [35]. This enables an uncontrolled but state-dependent cation flux, termed gating pore or *ω*-currents, which leads to an ion imbalance and pathology (for review see [36]). Several Cav1.1 pathological variants were already biophysically characterized. Out of seven measured periodical paralysis-causing arginine substitutions, five elicited *ω*-currents *in vitro* [32,37–40], two further have not been assessed for their *ω*-current capability and one harbored a substitution of a negative counter charge in III-S3 [41]. Recently, another L-type calcium channel, Cav1.3, has been shown to elicit *ω*-currents *in vitro* when arginine 990 was substituted by histidine [35].

In this study, we characterized the effects of two Cav1.4 substitutions, Arg964Gly (RG) and Arg1288Leu (RL), *in vitro*, both of which have been found in patients diagnosed with AIED [42]. We aimed to elucidate the effect of these missense mutations on protein expression and function. While the total channel expression and stability was unaffected in both, RG and RL according to statistical analyses, the current densities were decreased, presumably caused by a lower single-channel conductance. Homology modeling also suggested the loss of salt bridges, implying a reduced response to changes of voltages across the membrane likely explaining the alterations in biophysical properties compared to wild type. For RL, water wires were hypothesized by structural modeling, enabling *ω*-currents. Thus, our data provide the first possible indication of *ω*-currents in a pathological variant of Cav1.4.

## Experimental procedures

### Cloning of mutant Cav1.4 channels

Both CSNB2-associated Cav1.4 mutations Arg964Gly and Arg1288Leu [42], there referred to as p.(Arg975Gly) and p.(Arg1299Leu); UNIPROT O60840–1) were inserted into the “pCI mammalian expression vector” (E1731, Promega) by applying splicing by overlap extension PCR. In short, the human Cav1.4 coding sequence (JF701915) acted as a template for the first round of PCR for which overlapping forward and reverse primers were designed, carrying a codon optimized region of change – the 2890–2892 nt region was changed from CGG to GGC (Arg → Gly), the 3862–3864 nt region was altered from CGA to CTG (Arg → Leu). 5’ and 3’ flanking primers harbored the restriction enzyme recognition sites NheI and SalI, respectively. The resulting PCR fragments were combined in a second round of PCR, adding the flanking primers. The final full-length PCR fragment was inserted into the pCI backbone, both digested with NheI and SalI restriction enzymes (step 1). For detection in immunoblotting experiments, the same cloning strategy and primers have been applied to clone plasmids, taking an HA-tag-carrying human Cav1.4 coding sequence (CDS) as a template, in which the tag is inserted after nucleotide 2037 in the S5-S6-loop of domain II [43] (step 2). Sequence identity was confirmed by sequencing (Primers used in steps 1 and 2 are indicated in SupplementaryFigure 1).

### Cell culture and transfection

Both, tsA-201 (immunoblotting;#96121229, ECACC) and HEK-293 (whole-cell patch-clamp recordings [44];) cells were cultured in Dulbecco´s modified Eagle´s medium (DMEM, R15–801, PAA) supplemented with 10% fetal bovine serum (10270–106, Gibco), 2 mM glutamine (25030–032, Gibco), 10 units/ml Penicillin (Cat# *P*-3032, Sigma-Aldrich), 10 µg/ml Streptomycin (Cat# S-6501, Sigma-Aldrich) and maintained at 37°C and 10% CO_2_. Cells were grown and split at 80% confluency using PBS-EDTA and trypsin (25030–032, Gibco) for cell dissociation. The passage number did not exceed 18 passages in tsA-201. Cells were transiently transfected via calcium phosphate (CaPO_4_) precipitation as described previously by Burtscher [21].

### Protein preparation and immunoblotting

HA-tagged wild type (WT), Cav1.4-Arg964Gly (RG) or Cav1.4-Arg1288Leu (RL) were expressed together with their α_2_δ-_1_ and β_3_ subunits in tsA-201 cells, seeded in 10 cm cell culture dishes (confluency 70–80%). As a negative control, a Cav1.4 construct without HA tag was taken. Protein preparation, SDS-PAGE and western blot carried out as by Hofer [45] with following modifications. For protein concentration measurement, Bio-Rad’s “Protein Assay Reagent Concentrate” (5000006) was mixed with protein solution for subsequent photometric quantification at 595 nm. The HA-tag was targeted with anti-HA high-affinity antibody (1:1000, #11867423001, Roche diagnostics GmbH), tubulin with anti-ɑ-tubulin (1: 25 000, CP06, Merck) and mEmerald with anti-GFP (1:15 000, A6455, Thermo Fisher Scientific). Peroxidase-conjugated goat anti-mouse (1:8 000, 31430, Thermo Fisher Scientific), goat anti-rabbit (1:15 000, A0545, Merck) or goat anti-rat (1:10 000, 112-035-003, Jackson ImmunoResearch) have been applied as secondary antibodies.

### Cycloheximid chase experiments

For cycloheximide (CHX) chase experiments, tsA-201 cells (2.2e + 06 cells per 100 mm dish) were seeded and transfected as described. Cells were treated two days after transfection with 20 μg/ml CHX (C1988, Merck) for 2, 4 and 8 h. Immunoblotting was carried out as described above with the following changes: 15 µg of extracted membrane proteins was loaded and detection of the Na/K-ATPase (1:100 000; #ab76020, Abcam) was preferred instead of alpha-Tubulin. Due to space restrictions, WT samples of one transfection were used on two separate plots for comparison with RG and RL protein levels. Thus, the mean of the resulting WT signal intensities after normalization to loading control over these two blots served as one value for WT samples.

### Electrophysiological recordings

HEK-293 cells, stably expressing α2δ-1 and β3 (Ortner et al., 2007), were transfected with plasmids, carrying the channel WT, RG or RL, re-plated the following day and patch-clamp experiments were performed two to three-day post transfection. All electrophysiological experiments were carried out at room temperature. Electrodes were pulled from glass capillaries (borosilicate glass, Harvard Apparatus, Cat. Num. 64–0792) using a micropipette puller (Sutter Instruments) and fire polished with an MF-830 Microforge (Narishige) with a final resistance of 1.5–3.5 MΩ. The intracellular solution contained (in mmol/L): 135 CsCl, 10 Cs-EGTA, 1 MgCl_2_, 10 HEPES, and 4 ATP-Na_2_ adjusted to pH 7.4 with CsOH. The bath solution contained (in mmol/L): 15 CaCl_2_ or BaCl_2_, 150 choline-Cl, 1 MgCl_2_, and 10 HEPES, adjusted to pH 7.3 with CsOH. Cells were recorded in the whole-cell patch-clamp configuration using Axopatch 200B Amplifier (Molecular Devices). Recordings were digitized (Digidata 1322A Digitizer, Molecular Devices) at 50 kHz (I-V, steady state inactivation protocols and non-stationary fluctuation analysis) or 20 kHz (5-s pulse), low-pass filtered at 2 kHz or 10 kHz (non-stationary fluctuation analysis), and subsequently analyzed using pClamp 10.2 software (Molecular Devices) and custom-made Matlab (The Mathworks Inc., MA, USA) script. Compensation was applied for 60–90% of the series resistance. Currents were leak subtracted online using P/4 subtraction or offline. The current-voltage relationships (I-V) were acquired by application of a 50-ms square pulse to different test potentials starting from a holding potential (HP) of −89 mV. I – V curves were fitted to the equation:

$$I=\frac{G_{max}*\left(V-V_{rev}\right)}{1+e^{\frac{V_{0.5}-V}{k}}}$$

where V_rev_ is the extrapolated reversal potential, V is the test potential, I is the peak current amplitude, G_max_ is the maximum slope conductance, V_0.5,act_ is the half maximal activation voltage and k is the slope factor. The activation time constant tau (α) and the time to peak were extrapolated by fitting with mono-exponential function to the rising phase of each I-V sweep. The voltage-dependence of activation was obtained by calculating the conductance at each voltage-step and comparing them to the maximal conductance G_max_:

$$G=-\frac{I*1000}{V_{rev}+V}$$

The sweep at the reversal potential, where no net current flow was observed, was used to measure the ON-gating charge (Q_ON_), by integrating the first 2 ms of the test pulse. Steady-state inactivation was assessed by calculating the ratio between current amplitudes of a 50 ms test pulse to V_max_ before and after holding cells for 5 s at various conditioning test potentials. The steady-state inactivation curves were analyzed employing the following Boltzman relationship:

$$I=I_{SS}+\frac{1-I_{SS}}{1+e^{V-\frac{V_{0.5,inact}}{k_{inact}}}}$$

I_ss_ is the non-inactivating fraction and k_inact_ is the slope factor. Ion current inactivation was determined by calculating the ratio between the peak and the residual current after 250 ms (r250) applying 300 ms square pulse at different potentials (Δ10 mV) using 15 mM Ca^2+^ or Ba^2+^ as charge carrier. To investigate the inactivation kinetics of the channels, we pulsed the cells to V_max_ for 5 s.

Non-stationary fluctuation analysis (NSFA) was applied to calculate single-channel currents as well as to quantify channels expressed in the cell surface and the open probability. As a guide, the following tutorial was used [46]. Here, cells were depolarized from a HP of − 79 to+41 mV for 10 or 50 ms to maximally activate the channels before stepping back to −49 mV for 10 ms. 3 µM BayK8644 (BayK) was used during NSNA recordings to increase the channels opening time. The mean current and variance of 250–500 traces were calculated. To prevent capacitive transient interference, we started the analysis shortly after the peak tail current reached its maximum. The variance as a function of the mean current resulted in a parabolic curve that could be fitted by the following equation:

$$\sigma^{2}=i*I-\frac{I^{2}}{N}+b$$

where σ^2^ is the variance of the mean tail current, i the single channel current, I the mean tail current, N is the number of channels, and b is the background fluctuation described by the variance offset. To calculate the single conductance, the derivative of the prior function needed to be taken in order to quantify the smallest alteration in variance.

$$\frac{d\sigma^{2}}{dI}=i-\frac{2I}{N}$$

When all channels are open, we can substitute i*N for I, solving the function and computing -I and thereafter N. Opening probability P_0_ was assessed by comparing the maximum mean currently measured against the theoretical maximal mean current. The number of channels per cell was normalized to the cell size assuming a capacitance of 1 μF/cm^2^.

### Homology modeling

Structures of the WT Cav1.4 VSD III and VSD IV, and the two mutant channels RG and RL were modeled in the activated/inactivated and in the resting state by generating homology models based on the available high-resolution cryo-electron microscopy (EM) structures of voltage-gated Cav and Nav pore-forming subunits [47–49]. The following structures were used as templates: inactivated state: Cav1.1 α1-subunit structure (PDB accession code: 5GJV); resting state: NavAb disulfide crosslinked mutant α-subunit (PDB accession code: 6P6W).

Homology modeling has been performed using Rosetta and MOE (Molecular Operating Environment, version 2020.09, Molecular Computing Group Inc., Montreal, Canada). Additionally, ab initio Rosetta was used to model structures for loops that were not resolved in the original templates. The structures for the mutations were derived from the respective wild-type model by replacing the affected amino acid residue followed by a local energy minimization using MOE. The C-terminal and N-terminal parts of each domain were capped with acetylamide (ACE) and N-methylamide to avoid perturbations by free charged functional groups. The structure models were embedded in a plasma membrane consisting of POPC (1-palmitoyl-2-oleoyl-sn-glycero-3-phosphocholine) and cholesterol in a 3:1 ratio, using the CHARMM-GUI Membrane Builder. Water molecules and 0.15 M KCl were included in the simulation box. Energy minimizations of WT, RG, and RL structures in the membrane environment were performed. The topology was generated with the LEaP tool of the AmberTools20, using force fields for proteins and lipids, ff14SBonlysc, and Lipid14, respectively. All structures were gradually heated from 0 to 300 K in two steps, keeping the lipids fixed, and then equilibrated over 1 ns. Then molecular dynamics simulations were performed for 500 ns, with time steps of 2 fs, at 300 K and in anisotropic pressure scaling conditions. Van der Waals and short-range electrostatic interactions were cut off at 10 Å, whereas long-range electrostatics were calculated by the Particle Mesh Ewald (PME) method. PyMOL was used to visualize the key interactions and point out differences in the wild-type and both mutant structures (The PyMOL Molecular Graphics System, Version 2.0 Schrödinger, LLC).

To quantify the number of water molecules in the wildtype and substituted VSD III and VSD IV, we calculated water density profiles [50]. We used the positions of water molecules along the z-axis perpendicular to the membrane plane over 300 ns simulations. To quantify flexibility of the S4 helix, we calculated the B-factor based on the Cα atoms by using cpptraj [50]. Also, Cα-Cα distances have been calculated with cpptraj. Additionally, we calculated the secondary structure content with cpptraj.

### Statistics

All values are presented as means ± SEM for the indicated number of experiments (n). For comparisons of two groups, data were analyzed by unpaired t-test or Mann-Whitney U-test, depending on whether our data were normally distributed. One-way ANOVA with Bonferroni post hoc or Kruskal-Wallis with Dunn’s post hoc test was used to test the statistical difference between more than two groups. Statistical significance was set at *p* < 0.05. A power analysis was performed in Sigmaplot 14.5 to estimate the required sample size.

## Results

### Structural characteristics and expression of two gating charge neutralizing Cav1.4 variants

**Figure 1.**
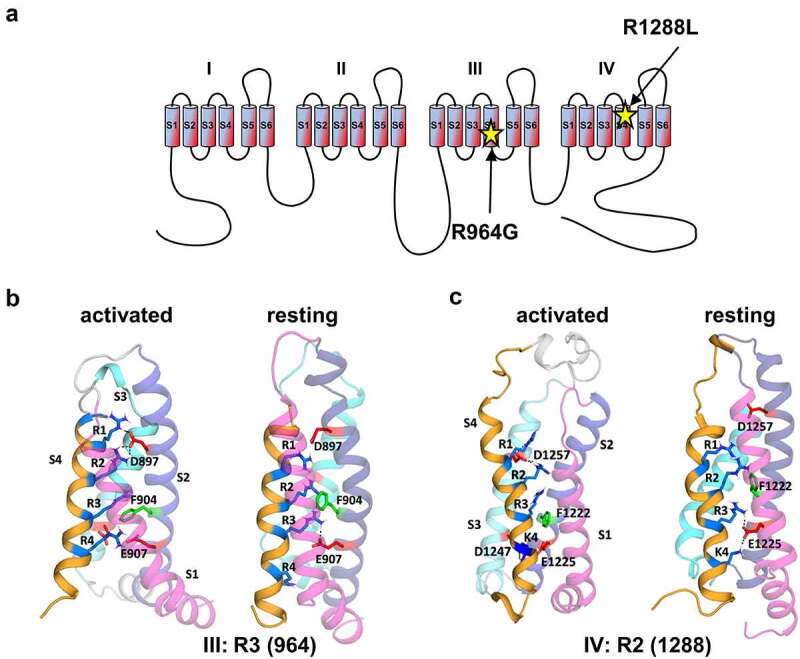
Cav1.4 amino acid substitutions in voltage sensor domains III and IV. (A) Location of R964G and R1288L substitution sites in the third (III) and fourth (IV) S4 voltage-sensing domain of Cav1.4, respectively (UNPROT O60840–2). (B) Homology model of voltage sensor domain III: whereas in its active conformation (left), Arginine 3 (R3, 964) in the III-S4 helix R3 did not interact with negative counter charge, R3 formed a salt bridge with E907 in the channel resting state (right). (C) Homology model of voltage sensor domain IV: while Arginine 2 (R2, 1288) in the IV-S4 helix formed a salt bridge with D1257 in the channel active state (left), R2 did not interact with negative counter charges in the channel resting state (right).

Two previously reported missense mutations in the coding sequence of Cav1.4 that lead to the substitution of Arg964 by glycine (RG) or Arg1288 by leucine (RL) have first been described in a Danish retrospective single-center study [42]. Both arginines are located in the voltage sensor (S4 helix) of the channel’s voltage-sensing domain (VSD) (Figure 1A). They belong to a group of positively charged amino acids in this helix, which are crucial for sensing electrical field changes and relocate accordingly to open and close the α1 pore. In the channels’ activated state, three arginines (R1, R2, and R3) are located above the hydrophobic constriction side. In the resting state model, we find that R2 is at the same height as the conserved phenylalanine residue, which is one of the most important residues within the hydrophobic restriction site [51,52] (F904; Figure 1B).

Looking closer on the molecular details, homology modeling predicted a supporting role of R964 in the resting state due to an interaction with the negative counter charge E907 in the same domain, thus, stabilizing the voltage sensor (Figure 1B). On the other hand, R1288 is indicated to stabilize the S4 helix in the activated state by forming a salt bridge with D1257 (Figure 1C). Both amino acid substitutions, RG and RL, would neutralize the positive charge in the S4 helix in domains III and IV, respectively. This could lead to the loss of interaction with corresponding negative counter charges and a destabilization of the S4 helix in the membrane.

**Figure 2.**
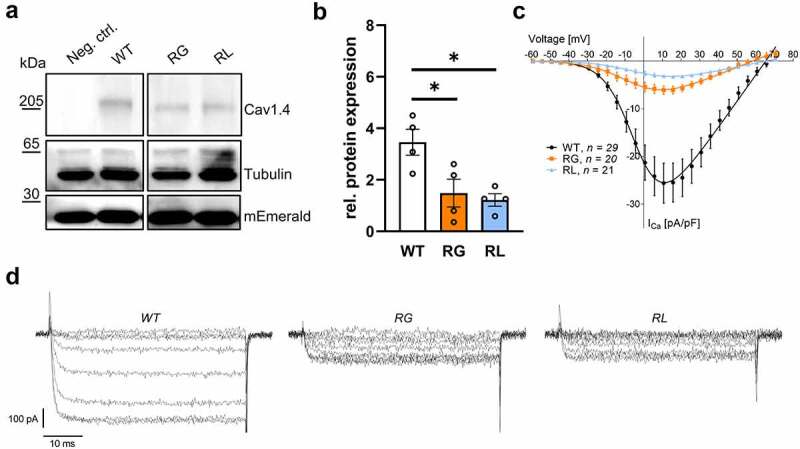
Neutralization of gating charges reduced functional channel expression. (A) Representative western blot of wild type (WT) compared to Cav1.4-R964G (RG) and Cav1.4-R1288L (RL) channels expressed in tsA-201 cells. As negative control (neg. ctrl.) were cells expressed Cav1.4 that lacked an HA-tag. (B) Mean protein expression after four independent transfections normalized to mEmerald levels: WT (3.46 ± 0.50), RG (1.84 ± 0.54; *p* = 0.0366) and RL (1.22 ± 0.24; *p* = 0.0186). Data are shown as mean ± SEM. Statistical analysis: One-way ANOVA with Bonferroni post-hoc test; *p < 0.05. (C) Current densities of WT, RG and RL heterologously expressed in HEK-293 cells. 15 mM Ca2+ was used as charge carrier. (D) Representative Ca2+ currents mediated by of WT, RG and RL channels. Data are shown as mean ± SEM for the indicated number of experiments. Statistical analyses: see table 1.

Destabilization of the S4 helix could lead to altered biogenesis, resulting in changes in total protein expression as shown in potassium channels [53]. To test this hypothesis, we co-expressed α_1_, α_2_δ-_1_ and β_3_ subunits in tsA-201 cells together the fluorescent protein mEmerald to account for variations in transfection efficiency and allow for normalization to an exogenous protein (Figure 2A). RG levels reached 42% and RL 35% of that of wild type (WT) channel expression (Figure 2B), showing a significant decreased protein abundance in the membranous fraction. This finding is in accordance with the reduced Ca^2+^ and Ba^2+^ current density in RG or RL channels; with the strongest also attributed to RL (Figure 2C and D, SupplFig. 1 (Ba^2+^), Table 1).

**Table 1.** Biophysical parameters and statistical comparison of wild type (WT), Cav1.4-R964G (RG) and Cav1.4-R1288L (RL). Abbreviations: CD, current density; V_0.5,act_, half maximal voltage of activation; k_0.5,act_, steepness of activation curve; V_max_, voltage of maximal current density; act thresh, voltage at 5% of current density; V_rev_, reversal potential; V_0.5,inact_, half maximal voltage of inactivation; k_0.5,inact_, steepness of inactivation curve. 15 mM calcium or barium was used as charge carrier. Data are presented as mean ± SEM. Statistical analysis: One-way ANOVA with Bonferroni post hoc or Kruskal-Wallis with Dunn’s post hoc test.

Ca^2+^	WT	RG	RL
CD [pA/pF]	25.99 ± 4.18, *n* = 29	6.20 ± 0.80, *n* = 20, *p* = 0.0003	3.21 ± 0.33, *n* = 21, *p* < 0.0001
V_0.5, act_ [mV]	1.48 ± 0.39, *n* = 29	2.63 ± 0.98, *n* = 20, *p* = 0.2321	7.88 ± 0.82, *n* = 21, *p* < 0.0001
k _act_	8.69 ± 0.17, *n* = 29	10.42 ± 0.24, *n* = 20, *p* < 0.0001	10.66 ± 0.31, *n* = 21, *p* < 0.0001
V_max_ [mV]	13.31 ± 0.34, *n* = 29	13.47 ± 0.73, *n* = 20, *p* = 0.5547	19.00 ± 0.509, *n* = 21, *p* < 0.0001
act thresh [mV]	−32.85 ± 0.71, *n* = 29	−40.58 ± 1.48, *n* = 20, *p* < 0.0001	−36.31 ± 1.22, *n* = 21, *p* = 0.0525
V_rev_ [mV]	60.25 ± 0.83, *n* = 29	55.12 ± 1.98, *n* = 20, *p* = 0.0112	61.66 ± 1.82, *n* = 21, *p* = 0.4540
V_0.5, inact_ [mV]	−16.00 ± 1.05, *n* = 8	−12.07 ± 2.89, *n* = 10, *p* = 0.2624	−7.49 ± 1.51, *n* = 11, *p* = 0.0005
k _inact_	−6.73 ± 0.66, *n* = 8	−10.94 ± 1.21, *n* = 10, *p* = 0.0119	−11.49 ± 2.74, *n* = 11, *p* = 0.0006
**Ba2+**			
CD [pA/pF]	20.64 ± 3.20, *n* = 19	5.90 ± 0.86, *n* = 27, *p* < 0.0001	2.53 ± 0.38, *n* = 12, *p* = 0.0001
V_0.5, act_ [mV]	−9.67 ± 0.62, *n* = 19	−8.75 ± 1.12, *n* = 27, *p* = 0.5220	−2.06 ± 2.16, *n* = 12, *p* = 0.0003
k _act_	7.74 ± 0.12, *n* = 19	9.71 ± 0.19, *n* = 27, *p* < 0.0001	10.21 ± 0.33, *n* = 12, *p* < 0.0001
V_max_ [mV]	1.31 ± 0.65, *n* = 19	2.07 ± 0.33, *n* = 27, *p* = 0.6855	6.70 ± 1.06, *n* = 12, *p* = 0.0002
act thresh [mV]	−38.26 ± 0.73, *n* = 19	−48.55 ± 0.72, *n* = 27, *p* < 0.0001	−45.85 ± 0.85, *n* = 12, *p* < 0.0001
V_rev_ [mV]	43.25 ± 1.06, *n* = 19	42.80 ± 1.52, *n* = 27, *p* = 0.8001	44.08 ± 3.09, *n* = 12, *p* = 0.7657

### Unaltered protein stability in RG and RL

**Figure 3.**
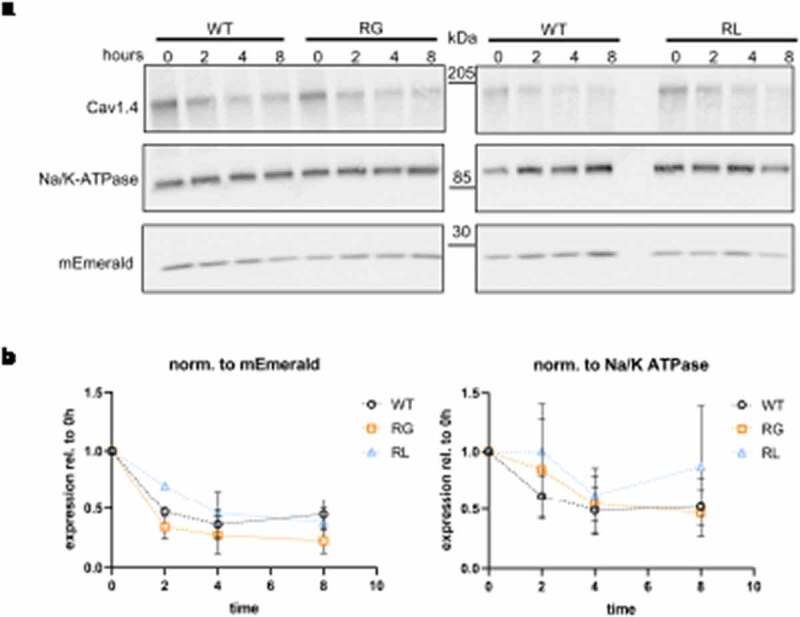
Cav1.4 gating charge mutations did not affect protein. (A) Representative western blot of wild type (WT), Cav1.4-R964G (RG) and Cav1.4-R1288L (RL) channels expressed in membranes of tsA-201 cells 0, 2, 4 and 8 hours after cycloheximide (CHX) addition. Co-expressed mEmerald and endogenous Na/K-ATPase have been used as loading control. (B) Channel expression levels were normalized to co-transfected mEmerald (left) or Na/K-ATPase expression (right) and their relative expression was displayed compared to 0 hour. Mann Whitney U test was applied at all time points resulting in no significant difference between WT and either of the two mutations. Data presented as mean ± SEM for three independent parallel transfections.

In addition to a decrease in cellular protein abundance, both amino acid substitutions could also lead to altered channel stability resulting in a reduction of current density. To test this possibility, cycloheximide (CHX) chase experiments were conducted. CHX is a translation inhibitor, thus is a compound that stops the synthesis of new proteins [54,55]. Like this, the level of proteins can be monitored over a time span and compared to its initial abundance [21]. Cav1.4 protein levels were normalized to Na/K-ATPase or co-transfected mEmerald, which were both stably expressed over the course of 8-hours post CHX addition (Figure 3A). At all timepoints examined, the relative channel levels were comparable for RG, RL, and WT channels (Figure 3B, Suppltable 1). Thus, these data do not indicate differences in protein stability due to substitution of the targeted arginine compared to WT.

### Changes in single channel properties

**Figure 4.**
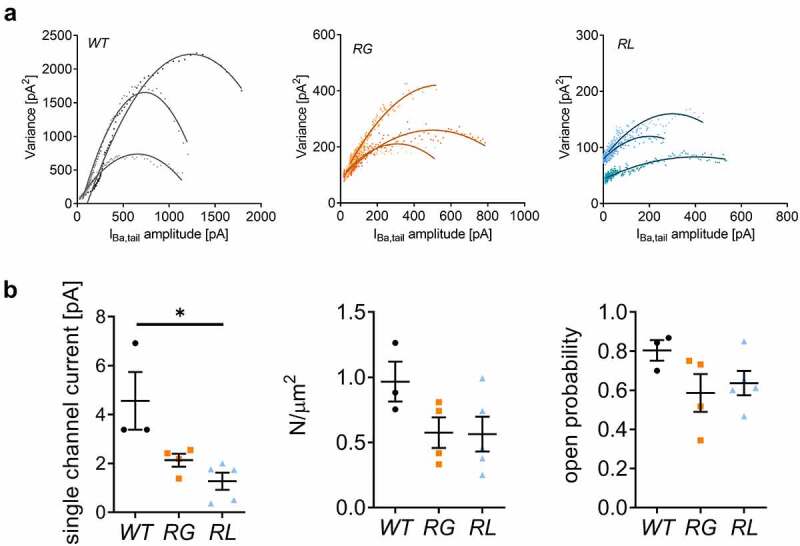
Mutations RG and RL reduced Cav1.4 single channel currents. (A) Exemplar variance was plotted against the mean current amplitude for wildtype (WT), Cav1.4-R964G (RG) and Cav1.4-R1288L (RL) (B) Left: single channel current for WT (4.56 ± 1.178), RG (2.13 ± 0.261, *p* = 0.0571) and RL (1.27 ± 0.347, *p = 0.0357). Middle: channel density on cell surface for WT (0.97 ± 0.153), RG (0.57 ± 0.118, *p* = 0.1143) and RL (0.56 ± 0.134, *p* = 0.1429). Right: open probability of WT (0.80 ± 0.052), RG (0.59 ± 0.097, *p* = 0.2286) and RL (0.64 ± 0.062, *p* = 0.1429). Data are given as mean ± SEM. Statistical analysis: Mann Whitney U test.

Besides channel quantity, also single-channel activity affects macroscopic ion currents. In this study, we performed NSFA to deduce single-channel properties, as well as open probability, and number of channels on the cell surface from the fluctuation observed in whole-cell recordings [21,46]. To do so, we activated the channels by depolarizing the cells to 41 mV and measured the tail current by a hyperpolarization to −49 mV. Because the open probability of Cav1.4 channels is low [21] we used measured channel activity in the presence of the calcium channel activator BayK. Collecting 250–500 sweeps of the tail current enabled us to calculate the variance from the mean at each time point after the peak of the tail-current (Figure 4A). The resulting parabolic profile is explained by the fact that there is no fluctuation in channel opening and closing when all channels are either opened or closed while variation is highest when 50% of the channels are opened. Calculating the smallest change in variation allows to acquire single-channel currents and also the total number of channels expressed in the plasma membrane. By knowing single-channel currents and quantity of channels, the maximum open probability can be determined [46]. Our data suggested that single-channel currents were significantly decreased in RL, reaching only 28% of WT levels. RG on the other hand, also elicited a smaller current (47% of WT); however, this difference did not reach statistical significance (Figure 4B, left). The number of channels expressed on the cell surface did not vary, and the open probability was also comparable to WT (Figure 4B, middle and right panel, respectively). These analyses implied that lower current densities in RG and RL might be due to a reduction in ions permeating single channels.

### Changes in the voltage-dependence of activation and inactivation

**Figure 5.**
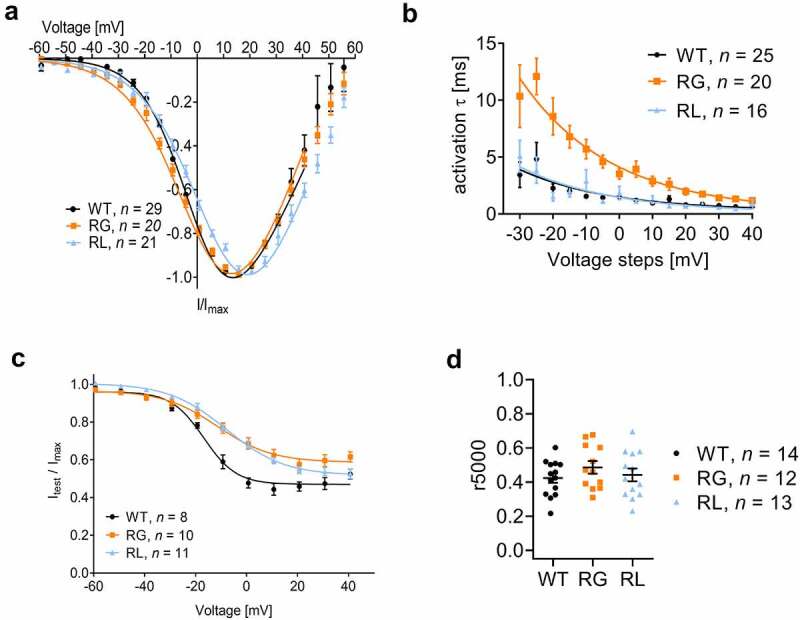
Cav1.4 gating charge mutations affected both voltage dependent activation and inactivation properties. Expression of wildtype (WT), Cav1.4-R964G (RG) and Cav1.4-R1288L (RL) channels in HEK-293. (A) I-V of calcium currents, normalized to currents at Vmax. (B) Time course of calcium current activation measured upon 50 ms depolarizing steps (I-V protocol). τ values, which came from the fit with exponential function, were plotted against their voltage. Mono-exponential function better described the course of activation for the majority of the traces. WT: 25 out of 29; RG: 20 out of 20; 16 out of 21. Data for the remaining traces that better fit by a bi-exponential fit were omitted. (C) Voltage-dependence of inactivation. For details see table 1. (D) Calcium current inactivation kinetics. Residual current after five second inactivation pulse to Vmax. Ca2+: WT (0.42 ± 0.028), RG (0.49 ± 0.037, *p* < 0.1794) and RL (0.44 ± 0.038, *p* = 0.6904). Data are given as means ± SEM. Statistics: unpaired t-test.

Seeing that S4 helices shape activation and inactivation properties of voltage-gated ion channels and neutralization of positive charges lead to changes in channel gating [32,37–40,56], we determined the functional properties of both channel variants in HEK-293 cells using whole-cell patch-clamp recordings. Both RG and RL activated at more hyperpolarized potentials and showed a decrease in the slope factor of the activation curve affecting the half maximal voltage of activation in RL but not RG channels (Figure 5A; Table 1 for Ca^2+^ and Ba^2+^ currents). We further characterized the activation time constants at different test potentials, as the destabilization of the S4 voltage sensor might change the kinetics of channel opening [40,57]. The opening of the RG channels slowed down compared to WT, an effect that was not seen in RL channels (Figure 5B). Our steady-state inactivation protocol elicited a significant increase in the slope factor causing a shift in the steady state inactivation to more positive voltages which was significant for RL but not for RG channels (Figure 5C, Table 1 for Ca^2+^ currents). We also compared the current at 40 mV as a measure of the plateau phase to evaluate inactivation properties and found a statistical difference in the remaining fraction between WT (0.46 ± 0.02) and RG (0.58 ± 0.03, *p* = 0.0063, unpaired t-test) but not compared to RL (0.51 ± 0.02, *p* = 0.1520, unpaired t-test) (Figure 5C). Current inactivation during a 5-s depolarizing test pulse to Vmax, showed no significant differences in the residual currents at the end of the test pulse (Figure 5D; SupplFig. 2B). One of the hallmarks of Cav1.4 channels, the lack of calcium-dependent inactivation (CDI), was seen in RG and RL compared to WT channels (SupplFig. 2C). Therefore, substitution of different arginines can have dissimilar effects on the biophysical properties of Cav1.4 channels.

### Reduction in gating current

**Figure 6.**
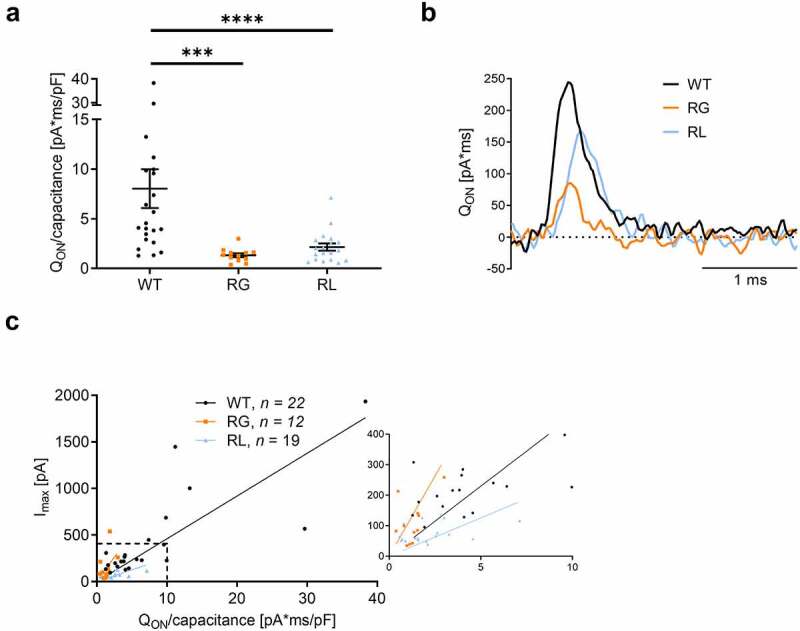
Gating currents were reduced in RG and RL channels. (A) ON-gating currents (QON) were measured at Vrev, where no net current flow was observed. QON current for each cell was normalized to its capacitance, in pA*ms/pF: WT 432.0 ± 99.2; RG 145.5 ± 40.9, ***p = 0.0007; RL 73.6 ± 7.3, ****p < 0.0001. Statistics: Mann Whitney U test. (B) Representative QON evoked by voltage pulses to the reversal potential, taken from the recordings shown in Fig. 2D. (C) Left, plot of Imax as a function of the total charge movement (QON) as in panel A. The inset on the right shows the area highlighted by the dashed line. Wildtype (WT), Cav1.4-R964G (RG) and Cav1.4-R1288L (RL). Slope: WT: 45.9 ± 5.4, RG: 105.2 ± 24.9 RL: 25.1 ± 3.8. Data are given as mean ± SEM.

We quantified gating currents because neutralizing one positive charge was previously shown to reduce upward movement of the S4 helix, which could alter coupling to the opening of the α1 pore [38]. We measured ON gating currents (Q_ON_) at the reversal potential and found a drastic reduction for both, RG and RL channels (Figure 6A–C). While this result could be explained also by a lower surface expression of the channel, our analysis of the non-stationary fluctuation (Figure 4B) and Cav1.4 gating charge mutations affected both voltage-dependent activation and inactivation properties. Expression of wildtype (WT), Cav1.4-R964G (RG) and Cav1.4-R1288L (RL) channels in HEK-293. (A) I-V of calcium currents, normalized to currents at Vmax. (B) Time course of calcium current activation measured upon 50 ms depolarizing steps (I-V protocol). τ values, which came from the fit with exponential function, were plotted against their voltage. Mono-exponential function better described the course of activation for the majority of the traces. WT: 25 out of 29; RG: 20 out of 20; 16 out of 21. Data for the remaining traces that better fit by a bi-exponential fit were omitted. (C) Voltage-dependence of inactivation. For details see Table 1. (D) Calcium current inactivation kinetics. Residual current after 5-s inactivation pulse to Vmax. Ca2+: WT (0.42 ± 0.028), RG (0.49 ± 0.037, *p* < 0.1794), and RL (0.44 ± 0.038, *p* = 0.6904). Data are given as means ± SEM. Statistics: unpaired t-test. Chase experiments (Figure 3B) hint at a comparable channel expression. Plotting the normalized Q_ON_ against the cell´s I_max_ (Figure 6C) implicated that the Q_ON_-I_Ca_ relationship followed different slopes: RL had a 2-fold decrease of the slope compared to WT, in good agreement with the significant smaller single-channel conductance (Figure 4B): The 2-fold increase of the slope of RG might, however, be an indication for efficient coupling between voltage gating and channel opening also reflected by the negative shift of the activation threshold (Figure 5A, Table 1).

### The possibility of ω-currents

**Figure 7.**
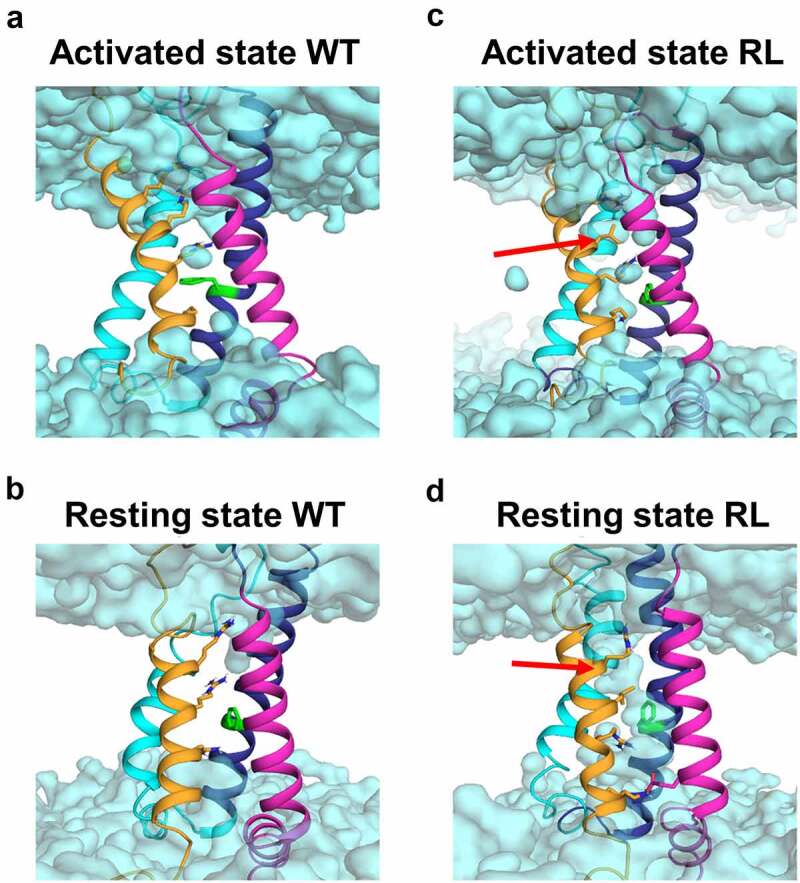
Mutation RL increased water occupancy in the voltage sensing domain IV. Homology modeling demonstrate a difference in water occupancy (cyan) in the VSD-IV activated (A and C) and resting (B and D) state between WT (A and B) and RL (C and D). The leucine in RL is indicated by a red arrow.

Numerous studies conducted on ion channels with S4-amino acid substitutions reported *ω*-currents, an additional, disease-driving ion-flux through one of the VSDs (for review see [36]). To test whether the substitution of RG or RL could enable *ω*-currents, we modeled the respective VSD in the activated and resting state. For the WT VSD III simulations, we found that only single water molecules can enter the hydrophobic constriction site; however, the gating charges (R1-R4) which formed salt-bridge interactions with negative counter charges in the S2/S3 helices (Figure 1C), prohibited the formation of a water wire (Figure 7A and B). Furthermore, interactions between the gating charges and the conserved phenylalanine residue contributed to sealing the hydrophobic core (Figures 1C and 7A and B).

For RG, MD simulations revealed an increase in flexibility of the S4 helix in the activated state as obvious by the increase in the B-factor and an unfolding of the lower part of the S4 helix in the resting state (SupplFig. 2). This unfolding was reflected in a loss of secondary structure content in the S4 helix by 15% and 35% in the activated and resting state, respectively. Due to these unfolding events, we could not reliably quantify the water molecules entering the VSD in RG channels. Yet, we found indications for state-dependent *ω*-currents for the RL VSD-IV. The substitution resulted in an increased water occupancy in the activated state (Figure 7C). In the WT activated state, R2 formed a stabilizing salt bridge interaction with D1257 (Figure 1B, dotted lines). Upon substitution to leucine, the volume occupied by the side chain of R2 decreased and the salt bridge interaction between D1257 and R2 was replaced by water molecules. Also, the Cα-Cα distance between RL and D1257 increased from 6.5±0.7 to 9.1±0.8 Å, indicating that the S4 helix moved away from the S3 and S2 helices, allowing for more water molecules to enter the VSD. We found an increase in water density in the RL activated state (Figure 7C), compared to the WT (water molecules: WT: 3.3±0.4, RL: 11.4±1.7). In the resting state of RL, we detected continuous water wires (Figure 7D), also reflected in a substantial increase of water molecules entering the hydrophobic gate (water molecules: WT: 2.5±0.7, RL: 9.3±1.1).

## Discussion

The characterization of disease-causing alterations in Cav1.4 channels is of interest to get a better understanding on how pathology might be caused by distinct mutations. In this study, we aimed to shed light on two pathological Cav1.4 variants, RG and RL [42], both of which lose a positive gating charge in their S4-helix. Such gating charge neutralizations in other voltage-gated ion channels are connected to diseases and have been characterized *in silico* and *in vitro* [32,35,37–40].

Our study determined channel membrane expression in a heterologous system because misfolding could be causing disturbances in biogenesis and faster degradation as shown with potassium channels carrying an amino acid substitution in a conserved S4 arginine [53]. We concluded that total protein expression in both arginine-substituted channels was significantly reduced compared to WT channels (Figure 2A and B). Additionally, Ca^2+^-current densities were also smaller in both RG and RL channels (Figure 2C). This reduction in current densities fits with data from HypoPP-causing Cav1.1-R1239H channels (RL analogue, IV-R2), in which the second arginine of VSD-IV is also affected and was a general observation in most of the tested L-type calcium channel mutations [32,35,37,40]. One explanation could be a reduced stability of Cav1.4 channels, as seen previously L860P Cav1.4 channels [21]. However, our CHX-chase experiments suggested that none of the amino acid substitutions lead to a faster degradation of the channel population compared to WT (Figure 3). This finding holds true as long as proteins with differential regulatory function for WT and pathological variants are not altered by the fact that CHX per se is cytotoxic as it disrupts the whole natural cellular physiology [58,59].

Nevertheless, a reduction in the current density can also be explained by a smaller single-channel conductance, less channel expression at the cell-surface and reduced channel open probability. Our NSFA data indicated a statistically significant decrease in single-channel Ba^2+^ currents for RL channels, as well as a trend toward a decrease in RG channels (Figure 4B). Even though the addition of BayK as channel activator could mask differences in channel open probability, we assumed that such effect might not be seen here because we knew from previous analyses that the fold-increase of WT and mutant Cav1.4 currents is different [21]. NSFA also indicated that channel densities in the plasma lemma were slightly, but not significantly, decreased in both mutations (Figure 4B center), fitting the western blot results (Figure 2).

Since to date no III-R3 substitution in voltage-gated calcium channels has been characterized, the only RG-analogous substitution is the HypoPP-causing mutations Nav1.4-R1135C/H [57]. Similar to RG (Figure 5A and C, Table 1), both substitutions in Nav channels elicited an increase in the slope of the activation and inactivation curve (as seen in RG and RL), whereas only Nav1.4-R1135H also slowed channel activation. Nav1.4-R1135C also caused a left-shift of the half maximal voltage of activation. Of note, a left-shift was also discovered in Cav1.1-R1239H (IV-R2), which would be the arginine substitute analogue in RL [37]. This finding is in contrast to our data, which indicate a right-shift of V_0.5,act_, V_max_ and V_0.5,inact_ for RL (Table 1). A right shift of voltage-dependence, however, was measured in Cav1.3- R990H (III-R3) [35] and Cav1.1-R900G (III-R2) [40]. These data tell us that each VSD and each arginine substitution should be examined individually to understand their functional consequences.

We also looked at the possibility of *ω*-currents, which were detected in Nav1.4-R1135C/H and numerous Cav1.1 channels (summarized here [40]:), as well as in Cav1.3 [35]. In our model of the WT VSD-IV in its activated and resting state, we found that water occupancy was reaching zero closer to the hydrophobic plug, thus no ions should be passing. Substitution of the second arginine to leucine resulted in the increase of water molecules in the VSD (Figure 7C and D), which could give rise to ion currents, especially in the closed state, in which R1 and R2 substitutions were shown to be prone for *ω*-currents. We also modeled the RG substitution in VSD-III but found that the glycine might break the S4 helix in open and resting state creating an undesirable environment for water occupancy measurements (not shown). This loss of integrity might explain the slow activation of the α1 pore as tight coupling is missing, while increasing the flexibility of the S4 helix (Suppl.fig. 2).

Overall, RG and RL channels have lost functionality as Ca^2+^ influx is reduced. In an *in vivo* mouse model expressing non-conducting Cav1.4, Maddox et al. found that the lack of Ca^2+^ signaling was shown to still generate the proper molecular construction of the rod axon terminals [60]. Postsynaptic partners, however, were inefficiently recruited. A similar, but less drastic effect might occur at those synapses as some Ca^2+^ is still able to be conducted. Also, the nob2 mouse, in which an N-terminally cleaved Cav1.4 channel is expressed at low abundance, showed aberrant synaptic signal transmission apparent by reduced ERG b-waves but ribbons were still mostly preserved and elongated [61,62]. An additional effect, driving Cav1.4 mediated retinal diseases like CSNB2 or AIED, could be the hyperpolarization shift of activation as seen in Cav1.4-I745T mice [3,63–65]. WT channels utilize the full voltage range of photoreceptors while the observed reduction in current density in both RG and RL channels significantly reduces the change in Ca^2+^ influx within this range [14]. Moreover, the activation threshold of RG substituted channels is significantly left shifted compared to WT, suggesting that Ca^2+^ dynamics might even be more reduced due to the higher Ca^2+^ influx at hyperpolarized potentials (Figure 5A; please note that the voltage dependence of activation and inactivation was shifted by 15 mV to positive voltages under our recording conditions; 15 mM Ca^2+^ as charge carrier [18]). In RL, the change in Ca^2+^ dynamics might rather be due to the difference in current density because the fraction of current activated within the physiological voltage range of a photoreceptor was similar to WT (Figure 5A; fraction of current activated was 1% (WT), 1.3% (RG), and 1.8% (RL) and 18.4% (WT), 25% (RG), and 18.9% (RL) at −55 and −20 mV, respectively).

## Conclusion

We biophysically characterized Cav1.4 RG and RL channels *in vitro*. The changes in their functional properties could account for defects in synapse development due to reduced Ca^2+^ signaling thereby affecting signal transmission. To the best of our knowledge, this is the first study that adds *ω*-currents to the equation of pathological factors in addition to channel gating changes. *ω*-currents would add one further retinal stressor, very likely changing the fine-tuned synaptic environment and axon terminal physiology. A limitation of our latter conclusion is that we deduce *in vivo* implications by interpreting *in silico* and *in vitro* data which should be evaluated in a retinal environment. Specifically identifying the effect of possible *ω*-currents on the synaptic milieu would be of great interest in addition to developmental studies, which track synaptogenesis.

## Supplementary Material

Supplemental Material

## Data Availability

All data generated or analyzed during this study are included in this published article (and its Supplementary Information files). The data that support the findings of this study are available from the corresponding author, AK, upon reasonable request.
