# Supplementary material for: Characterization of two pathological gating-charge substitutions in Cav1.4 L-type calcium channels
Source: Channels (Austin). 2023 Mar 21;17(1):2192360. doi: 10.1080/19336950.2023.2192360 (PMC10038055; doi:10.1080/19336950.2023.2192360)
Supplement: Supplemental Material [file KCHL_A_2192360_SM7493.docx]

# Supplemental information

## Supplementary tables

Supplementary table 1: CHX-chase: Fraction of wild type (WT), Cav1.4-R964G (RG) and Cav1.4-R1288L (RL) 2, 4 and 8 hours post CHX addition compared to levels prior translation inhibition. Normalized to mEmerald (left) or Na/K-ATPase (right). Data are presented as mean ± SEM. Statistical analysis: Mann Whitney U test.

|  | mEmerald | | | |  | Na/K-ATPase | | |
| --- | --- | --- | --- | --- | --- | --- | --- | --- |
|  | WT | RG |  | RL |  | WT | RG | RL |
| 2h | 0.47±0.027 | 0.34±0.097 |  | 0.70±0.030 |  | 0.62±0.178 | 0.85±0.430 | 1.00±0.405 |
|  |  | p = 0.400 |  | p = 0.100 |  |  | p > 0.999 | p = 0.700 |
| 4h | 0.36±0.127 | 0.27±0.157 |  | 0.46±0.192 |  | 0.490±0.207 | 0.545±0.248 | 0.63±0.231 |
|  |  | p > 0.999 |  | p = 0.700 |  |  | p > 0.999 | p = 0.700 |
| 8h | 0.45±0.126 | 0.22±0.103 |  | 0.3800±125 |  | 0.52±0.251 | 0.48±0.202 | 0.87±0.517 |
|  |  | p = 0.400 |  | p > 0.999 |  |  | p > 0.999 | p > 0.999 |

## Supplementary figures

**Supplementary Figure 1.** Position of primers to verify sequence identify in the plasmids containing the coding sequences of RG and RL.


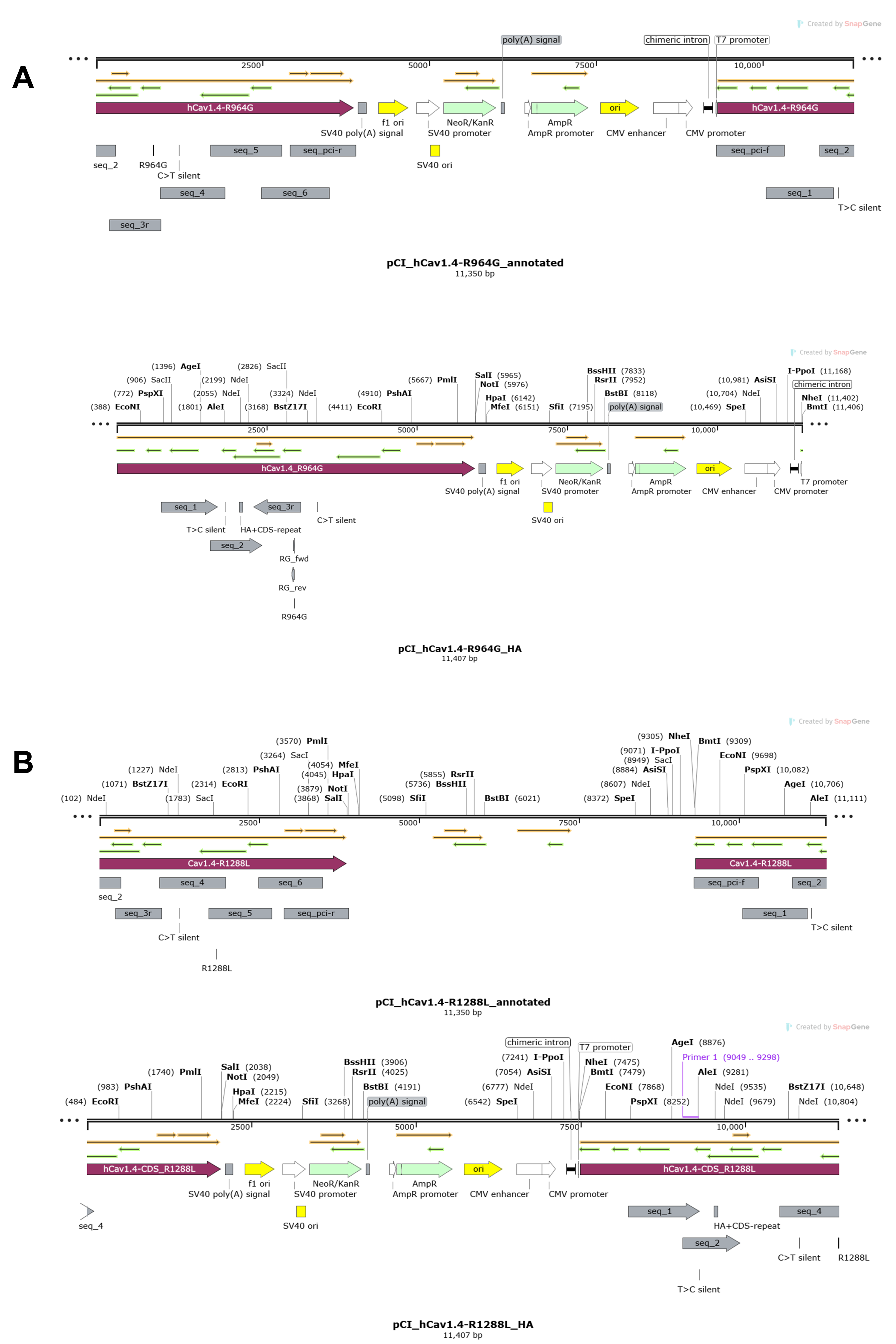


**Supplementary Figure 2:** The voltage dependence of current densities in transfected HEK-293 cells.

**
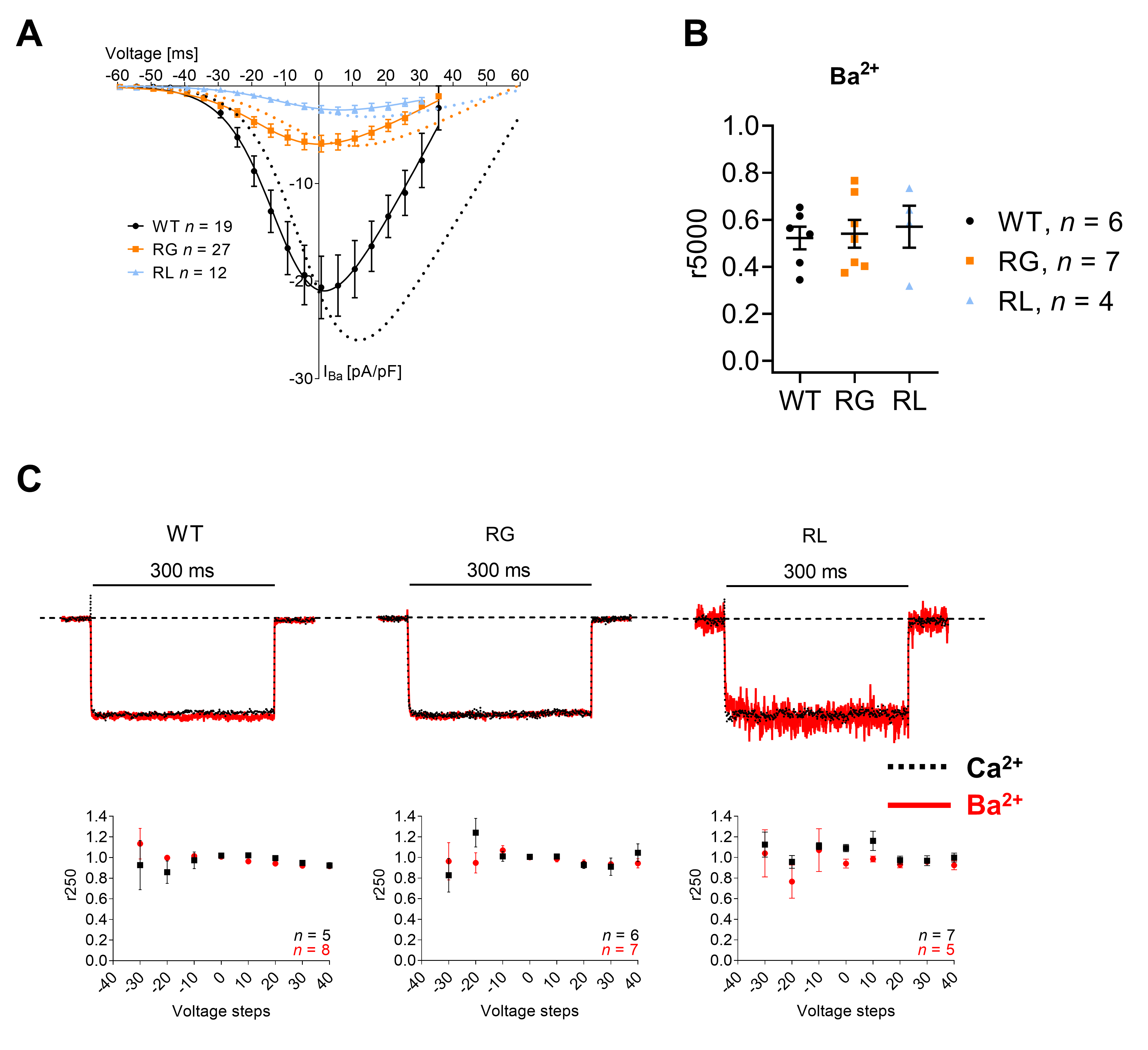
**

**Supplementary Figure 3:** S4 helix flexibility in wildtype (WT) and R964G VSD III.

**
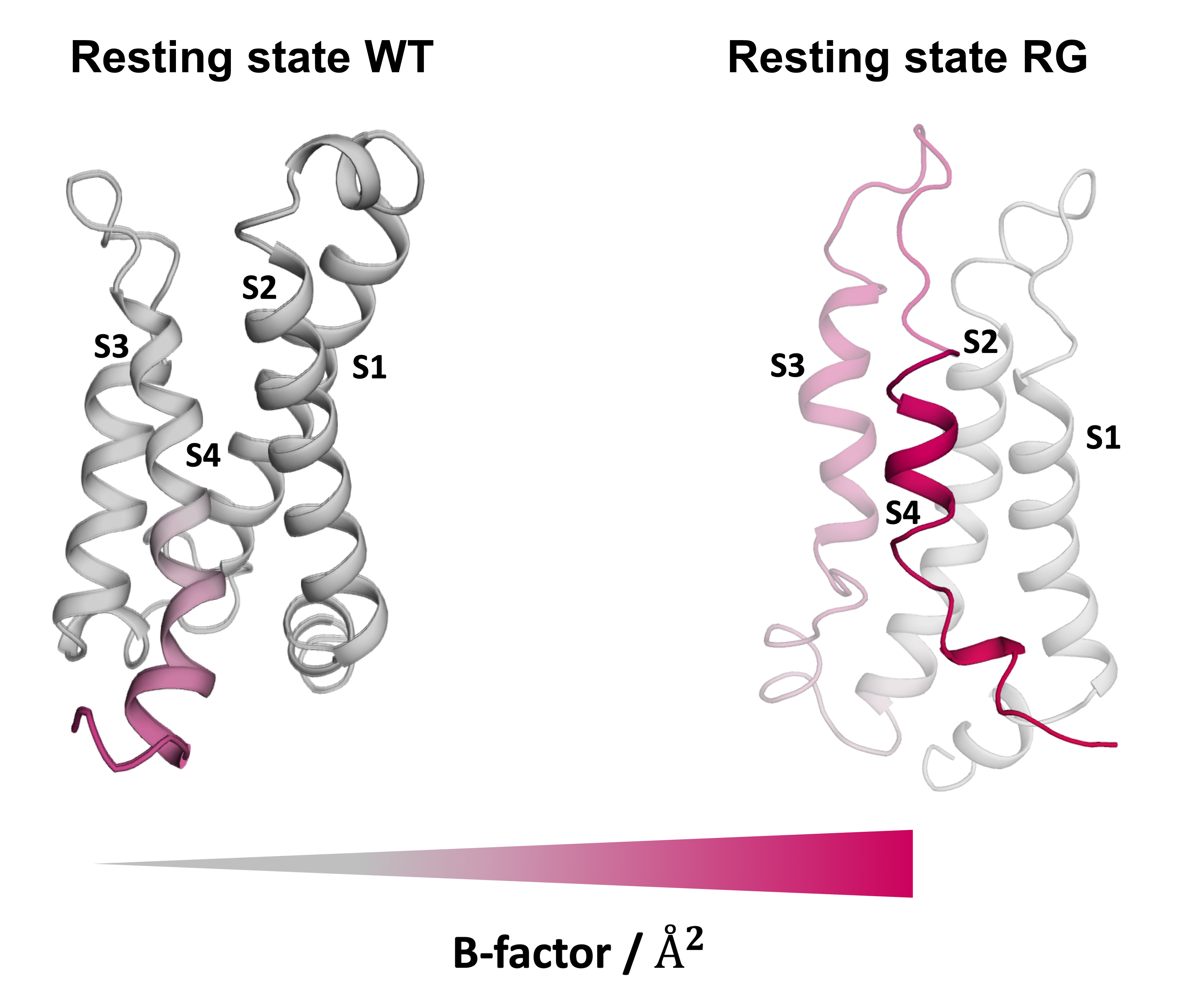
**

## Supplementary figure captions

**Supplementary Figure 1.** Position of primers to verify sequence identify in the plasmids containing the coding sequences of RG (A) and RL (B). The upper and lower parts show the sequencing primers used in step 1 and 2, respectively. For details see Methods section.

**Supplementary Figure 2:** The voltage dependence of current densities in transfected HEK-293 cells. (A) Current density of Wildtype (WT), Cav1.4-R964G (RG) and Cav1.4-R1288L (RL) at the voltages indicated are depicted. (B) Barium current inactivation kinetics. Ba^2+^: WT (0.52±0.048, N = 6), RG (0.54±0.059, N = 7, p<0.8217) and RL (0.57±0.089, N = 4, p=0.6202). (C) Calcium and barium current inactivation during a 300 ms test pulse to different potentials. Comparison of r_250_ values (ratio between peak current and current after 250 ms of depolarization).15 mM Ca^2+^ was used as charge carrier. Data are shown as mean ± SEM. Statistical analyses: for A and B see table 1. For C: unpaired Students t-test.

**Supplementary Figure 3:** S4 helix flexibility in wildtype (WT) and R964G VSD III. B-factor as metric for quantifying flexibilities mapped on the respective resting state structures. Areas of higher flexibility are depicted in red, while areas with low flexibility are shown in grey. The unfolding of the S4 helix is reflected in the non-helical parts of the S4.
